# Survival predictors in Vietnamese elderly AML patients treated with decitabine: Real-world evidence from a low- and middle-income country

**DOI:** 10.1016/j.lrr.2026.100565

**Published:** 2026-01-13

**Authors:** Ha Thanh Nguyen, Quoc Khanh Bach, Quoc Nhat Nguyen, Van Nam Nguyen, Thi Van Anh Nguyen, Hai Pham-The

**Affiliations:** a Department of Chemotherapy, National Institute of Hematology and Blood Transfusion, Hanoi, Vietnam; b Department of Hematology and Blood transfusion, Hanoi Medical University, Hanoi, Vietnam; c Department of Life Sciences, University of Science and Technology of Hanoi, Vietnam Academy of Science and Technology, Hanoi, Vietnam

**Keywords:** AML, Biomarkers, Decitabine, Elderly, Prognosis, Survival, Real-world

## Abstract

**Background:**

Hypomethylating agents such as decitabine represent a key treatment option for elderly acute myeloid leukemia (AML) patients who are unfit for intensive chemotherapy. However, real-world evidence from low- and middle-income countries (LMICs), including Vietnam, remains scarce. This study aimed to identify baseline clinical and laboratory predictors of 1-year overall survival (OS) in elderly Vietnamese AML patients treated with decitabine.

**Methods:**

This prospective, single-center, observational study was conducted at the National Institute of Hematology and Blood Transfusion, Vietnam, from April 2023 to June 2025. Seventy newly diagnosed AML patients aged ≥ 60 years received decitabine. The primary outcome was 1-year OS. Baseline demographic, clinical, hematologic, and immunophenotypic variables were analyzed using univariate and multivariate Cox regression. Treatment response was descriptively evaluated in patients completing ≥4 cycles.

**Results:**

The 1-year OS rate was 38.6 %, with a median survival of 276.0 days. Elevated bone marrow cell count (HR: 1.003, *p* = 0.002), fibrinogen (HR: 1.22, *p* = 0.044), and urea (HR: 1.23, *p* = 0.001) were independently associated with increased mortality. CD64 positivity (HR: 0.29, *p* = 0.029) and urban residence (HR: 0.43, *p* = 0.014) were protective. Among 39 patients completing ≥4 cycles, 48.7 % achieved a complete response. No significant survival difference was observed between responders and non-responders.

**Conclusion:**

This first prospective real-world study in Vietnam identified accessible predictors of survival in elderly AML patients receiving decitabine. The findings may aid early risk stratification using simple baseline parameters in LMIC settings, where access to molecular diagnostics may be limited, and warrant multicenter validation.

## Highlights

• Real-world study of elderly AML patients treated with decitabine in Vietnam.
• Five baseline variables independently predicted 1-year overall survival.
• Urban residence was associated with better survival than rural residence.
• CD64 positivity was independently linked to lower 1-year mortality.
• Bone marrow cell count identified as a novel prognostic marker.

## 1 Introduction

Acute myeloid leukemia (AML) is a clonal hematopoietic neoplasm of myeloid lineage characterized by the uncontrolled proliferation and accumulation of immature myeloid blasts in the bone marrow and peripheral blood, leading to acute bone-marrow failure and systemic manifestations. According to the World Health Organization (WHO) 2022 classification, AML is categorized into two major groups: *AML with defining genetic abnormalities*, which can be diagnosed irrespective of blast percentage (most cases may present with < 20 % blasts), and *AML defined by differentiation*, which requires ≥ 20 % myeloid blasts in bone marrow or peripheral blood, except for acute erythroid leukemia. Its incidence increases significantly with age, with a median diagnostic age of 68 years in the United States. Despite advances in therapy, outcomes in elderly AML remain poor owing not only to comorbidities and limited tolerance to intensive induction chemotherapy but also to adverse disease biology characterized by unfavorable cytogenetic and molecular features, as well as secondary or therapy-related ontogeny [1]. however, in many low- and middle-income countries (LMICs), including Vietnam, decitabine is preferred due to its wider availability, partial reimbursement under the national health insurance, and more convenient administration schedule.”

In recent years, hypomethylating agents (HMAs), such as azacitidine and decitabine, have become established alternatives for elderly patients unfit for intensive chemotherapy [2]; however, in Vietnam, decitabine is preferred due to its wider availability, partial reimbursement under the national health insurance, and more convenient administration schedule. Their efficacy has been demonstrated in several clinical trials and observational studies conducted in high-income countries with robust healthcare systems [3].

However, real-world evidence from LMICs remains limited, largely due to restricted access to diagnostic testing, variable availability of treatment and supportive care, and socioeconomic constraints that collectively affect treatment outcomes and data completeness. In these contexts, treatment decisions often rely on limited laboratory parameters rather than advanced molecular profiling, making real-world, practical data even more essential.

In LMICs such as Vietnam, where access to novel therapies like venetoclax is limited, decitabine continues to serve as the mainstay of treatment for elderly AML patients. Yet, prognostic markers, such as clinical, biochemical, or immunophenotypic, are poorly defined in these settings. Identifying accessible and low-cost predictors could improve risk stratification and treatment planning [2].

This study aimed to investigate baseline predictors of 1-year overall survival (OS) in elderly AML patients treated with decitabine in Vietnam. To our knowledge, it represents one of the first prospective, real-world evaluations of decitabine outcomes in a LMIC, addressing critical gaps in the literature and informing care in resource-limited environments.

## 2 Patients and methods

### 2.1 Patients

This prospective, single-center observational study was conducted at the National Institute of Hematology and Blood Transfusion (NIHBT), Hanoi, Vietnam, from April 2023 to June 2025. Eligible participants were newly diagnosed AML patients aged ≥ 60 years, deemed unfit for intensive chemotherapy and treated with decitabine (20 mg/m² IV daily for 5 consecutive days in 28-day cycles). Exclusion criteria included acute promyelocytic leukemia, severe hepatic/renal dysfunction, significant cardiac disease, contraindications to decitabine, administration of fewer than two cycles, or missing baseline data.

The study protocol was approved by the Hanoi Medical University Ethics Committee (871/GCN—HĐĐĐNCYSH-ĐHYHN), and written informed consent was obtained from all patients.

### 2.2 Definition

The primary outcome was 1-year OS, defined as survival status at 12 months after initiating decitabine. This time point was selected due to the high early mortality in elderly AML and its clinical relevance as an indicator of treatment effectiveness. Survival time was measured from treatment initiation to death or last follow-up. Cytogenetic risk was categorized as favorable, intermediate, or poor; patients with unavailable cytogenetic or molecular data were classified as unknown.

Treatment response and hematologic improvement (HI) were evaluated in patients completing four cycles of decitabine, based on the 2017 European LeukemiaNet (ELN) and 2006 International Working Group (IWG) criteria [4,5]. Four cycles were chosen as the response evaluation point, as decitabine generally requires ≥4 cycles to achieve efficacy [3], and few patients received additional cycles. Response categories included complete response (CR), CR with incomplete hematologic recovery (CRi), partial response (PR), and no response (NR).

### 2.3 Variables

Baseline demographic, clinical, laboratory, and immunophenotypic data were collected at AML diagnosis, including age, gender, region, medical history, clinical features, hematologic and biochemical parameters, bone marrow findings, immunophenotype, and cytogenetic risk. Outcome data (survival status and treatment response) were collected prospectively during follow-up.

### 2.4 Statistical analysis

Continuous variables were summarized as medians (IQRs), categorical variables as counts and percentages. Univariate Cox regression identified baseline variables for association with OS, with *p* < 0.20 as the threshold for inclusion in multivariate analysis. Variables with sparse data or unstable hazard ratios (HRs) were excluded. Clinically relevant predictors from prior AML literature were included regardless of univariate significance.

The final multivariate Cox model was built using bidirectional stepwise selection based on Akaike Information Criterion (AIC). Model assumptions were tested using Schoenfeld residuals, collinearity assessed via variance inflation factors (VIFs). Predictive performance was evaluated using C-index and Brier score, with internal validation via 10-fold and leave-one-out cross-validation (LOOCV). Kaplan–Meier analysis was used to estimate survival probabilities; adjusted survival curves were plotted for significant predictors. Continuous variables were stratified by quartiles. Statistical significance was set at two-sided *p* < 0.05.

## 3 Results

### 3.1 Patient characteristics

Seventy elderly AML patients treated with decitabine were included (Table 1). The median age was 65.5 years (IQR: 64.0 – 70.0), with 48.6 % male. A majority of patients (58.57 %) were from rural areas. Cytogenetic risk was intermediate in 50.0 %, favorable in 15.7 %, poor in 11.4 %, and unknown in 22.9 %. The most common French-American-British (FAB) subtypes were M2 (32.86 %) and M4 (30.0 %), with 32.86 % unclassified. Immunophenotyping showed high expression of CD33 (94.3 %), CD117 (84.1 %), MPO (81.4 %), CD13 (71.4 %), and HLA-DR (70.0 %). CD7, CD64, CD56, and CD38 were expressed in 70.0 %, 20.0 %, 17.1 %, and 15.7 %, respectively.

**Table 1:** Baseline clinical and laboratory characteristics of the study population.

**Characteristics**	
Age (years)	65.5 (64.0 – 70.0)
Gender Male Female	34 (48.6) 36 (51.4)
Residential area Urban areas Rural areas	29 (41.4) 41 (58.6)
Type of AML *De novo* Secondary (with MDS history)	62 (88.6) 8 (11.4)
FAB subtype M0 M2 M4 M5 M6 Unclassified	1 (1.4) 23 (32.9) 21 (30.0) 1 (1.4) 1 (1.4) 23 (32.9)
Cytogenetic risk Favourable Intermediate Poor Unknown	11 (15.7) 35 (50.0) 8 (11.4) 16 (22.9)
Anemia	68 (97.1)
Hemorrhage	35 (50.0)
Hepatomegaly	10 (14.3)
Splenomegaly	21 (30.0)
Weight loss	49 (70.0)
Hemoglobin (× 10⁹/L)	91.50 (79.75 – 102.0)
Reticulocytes (× 10⁹/L)	1.52 (1.0 – 2.14)
WBC (× 10⁹/L)	13.04 (4.21 – 54.80)
Platelet count (× 10⁹/L)	59.50 (32.00 – 106.50)
BMCc (× 10⁹/L)	104.45 (51.84 – 222.88)
Bone marrow blasts (%)	51.0 (30.0 – 79.75)
Peripheral blood blasts (%)	30.0 (8.50 – 70.0)
Urea (mmol/L)	6.20 (4.63 – 7.35)
Creatinine (μmol/L)	82 (71.75 – 100.50)
Uric acid (μmol/L)	325.0 (253.8 – 396.5)
Fibrinogen (g/L)	4.49 (1.49 – 5.35)
Lactate dehydrogenase (U/L)	651.0 (450.8 – 1033.0)
Aspartate aminotransferase (U/L)	25.0 (18.25 – 36.75)
Alanine aminotransferase (U/L)	21.50 (15.25 – 34.0)
Prothrombin time (s)	13.80 (12.72 – 15.07)
rAPTT (ratio)	1.13 (0.99 - 1.22)
D-dimer fragment	1415.0 (728.2 – 4129.0)
CD7 positive	14 (20.0)
CD13 positive	50 (71.4)
CD33 positive	66 (94.3)
CD38 positive	4 (5.7)
CD56 positive	11 (15.7)
CD64 positive	12 (17.1)
CD117 positive	61 (87.1)
HLA-DR positive	49 (70.0)
MPO positive	57 (81.4)

Note: Data are presented as median (interquartile range) for continuous variables and number (percentage) for categorical variables.

MDS: myelodysplastic syndrome.

BMCc: bone marrow total cell count (BMCc).

rAPTT: activated partial thromboplastin time ratio.

### 3.2 Treatment response

Patients received a median of 4 decitabine cycles (IQR: 2–6). Among 39 evaluable patients (≥4 cycles), the overall response rate (CR, CRi, or PR) was 58.9 %, with 48.7 % achieving CR and 10.3 % PR. HI was observed in 9 (23.1 %), of whom, 8 had erythroid and platelet response and 1 had platelet response alone. The clinical benefit rate (CR + CRi + PR + HI) was 82.1 %, while 17.9 % had no response. The median OS was 276.0 days (IQR: 112.20 – 395.20). The 30-day mortality rate was 5.7 %. Although patients with CR had numerically longer survival (median, 410.0 *vs*. 332.5 days), the difference was not statistically significant (*p* = 0.10).

### 3.3 Univariate analysis

Forty-three patients (61.4 %) died within 12 months; the 1-year OS rate was 38.6 %. In univariate Cox analysis (Table 2), urban residence predicted better survival (HR: 0.39; 95 % CI: 0.19–0.80; *p* = 0.010). Higher urea (HR: 1.15; 95 % CI: 1.04–1.28; *p* = 0.006), creatinine (HR: 1.01; 95 % CI: 1.00–1.02; *p* = 0.031), and lactate dehydrogenase levels (HR: 1.000; *p* = 0.046) were associated with increased mortality.

**Table 2:** Univariate Cox regression analysis of baseline variables and 1-year survival.

**Characteristics**	**HR**	**95% CI**	***p*-value**
Age	1.04	0.98 – 1.08	0.234
Gender Male Female	<REF>Reference 0.82	0.45 – 1.49	0.505
Residential area Urban areas Rural areas	0.50 Reference	0.26 – 0.95	0.034*
Type of AML De novo Secondary (with MDS history)	<REF>Reference 0.69	0.29 – 1.63	0.393
FAB subtype M2 M4 Others (M0, M5 and M6) Unclassified	0.98 Reference 0.48 1.01	0.46 – 2.09 0.06 – 3.69 0.53 – 2.32	0.958 0.958 0.791
Cytogenetic risk Favourable Intermediate Poor Unknown	0.72 0.82 Reference 1.14	0.22 – 2.36 0.31 – 2.18 0.39 – 3.28	0.588 0.691 0.811
Hemorrhage	1.53	0.84 – 2.81	0.168
Hepatomegaly	1.25	0.53 – 2.97	0.610
Splenomegaly	1.23	0.64 – 2.36	0.535
Weight loss	1.31	0.67 – 2.55	0.427
Hemoglobin	1.01	0.99 – 1.03	0.175
Reticulocytes	1.08	0.70 – 1.23	0.598
WBC	1.00	0.99 – 1.01	0.941
Platelet count	1.00	0.99 – 1.01	0.702
Bone marrow total cell count	1.00	0.99 – 1.00	0.181
Bone marrow blasts	1.00	0.99 – 1.01	0.507
Peripheral blood blasts	1.00	0.99 – 1.01	0.361
Urea	1.15	1.04 – 1.28	**0.006**⁎⁎
Creatinine	1.01	1.00 – 1.02	0.031*
Uric acid	1.00	0.99 – 1.00	0.249
Lactate dehydrogenase (LDH)	1.000	1.000 – 1.000	0.046*
Fibrinogen	1.09	0.92 – 1.32	0.303
Aspartate aminotransferase	1.01	0.99 – 1.02	0.246
Alanine aminotransferase	0.99	0.98 – 1.01	0.943
Prothrombin time	1.07	0.91 – 1.26	0.404
rAPTT (ratio)	1.28	0.35 – 4.68	0.707
D-dimer fragment	1.000	0.99 – 1.000	0.754
CD7 positive	0.79	0.36 – 1.69	0.537
CD13 positive	0.83	0.44 – 1.57	0.56
CD33 positive	1.29	0.31 – 5.37	0.719
CD38 positive	2.45	0.87 – 6.89	0.089
CD56 positive	1.27	0.56 – 2.85	0.567
CD64 positive	0.85	0.38 – 1.92	0.703
CD117 positive	0.56	0.25 – 1.25	0.156
HLA-DR positive	0.81	0.43 – 1.53	0.515
MPO positive	0.95	0.45 – 2.04	0.887

Note: HR: hazard ratio.

CI: confidence interval.

⁎ *p*-value < 0.05.

⁎⁎ *p*-value < 0.01.

**Fig. 1:**
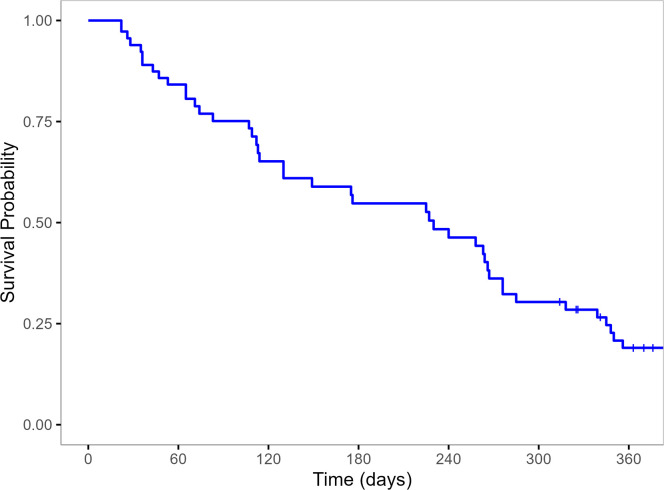
1-year Kaplan-Meier survival curve for the elderly AML population.

The 1-year Kaplan–Meier survival estimate was 37.6 % (95 % CI: 27.6–51.1 %), with a steady decline observed throughout follow-up (Fig. 1), supporting the relevance of 1-year OS as a prognostic endpoint.

### 3.4 Multivariate analysis

**Fig. 2:**
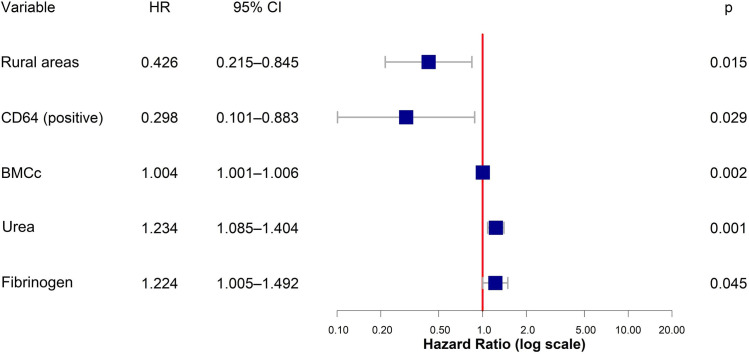
Multivariate Cox regression analysis for 1-year survival: HR (Hazard ratio), CI (Confidence interval), BMCc (Bone marrow total cell count), * p-value < 0.05, ** p-value < 0.01.

Variables with *p* < 0.2 in univariate analysis (excluding CD38 due to unstable HR), along with fibrinogen [6], peripheral blood blasts, WBC [7], and CD64 [8], previously reported as prognostic in AML, were included in the multivariate analysis. The final Cox model (Fig. 2) identified urban residence (HR: 0.43; 95 % CI: 0.21–0.85; *p* = 0.014) and CD64 positivity (HR: 0.29; 95 % CI: 0.10–0.88; *p* = 0.029) as protective factors. Higher BMCc (HR: 1.004; 95 % CI: 1.001–1.006; *p* = 0.002), fibrinogen (HR: 1.22; 95 % CI: 1.004–1.49; *p* = 0.044), and urea (HR: 1.23; 95 % CI: 1.08–1.40; *p* = 0.001) were associated with increased mortality.

**Fig. 3:**
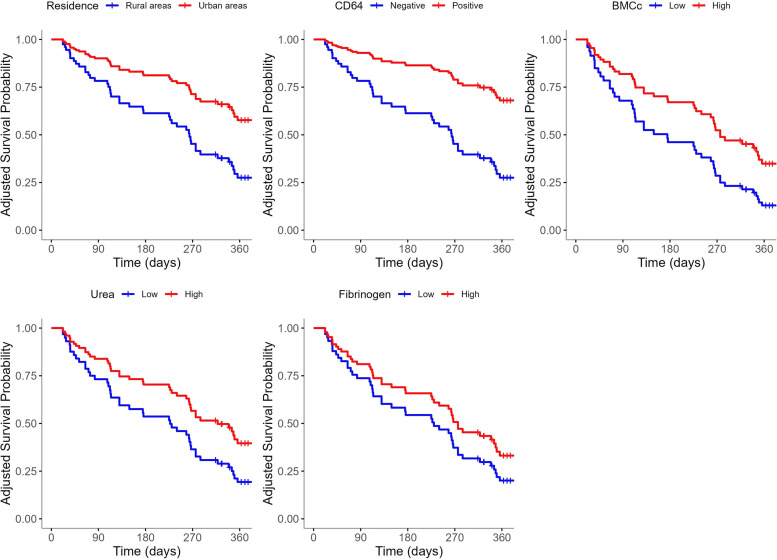
Adjusted 1-year survival curves from the multivariate Cox regression model by 5 significant predictors.

The model showed good fit (C-index: 0.675; SE: 0.045), satisfied proportional hazards assumptions (global *p* = 0.85), and reasonable predictive performance (*C*-index: 0.675, LOOCV Brier score: 0.206). Adjusted survival curves for significant predictors are presented in Fig. 3.

## 4 Discussion

This is the first real-world study in Vietnam to evaluate baseline survival predictors in elderly AML patients treated with decitabine. It highlights practical insights for AML management in resource-limited settings, where advanced diagnostics and novel therapies remain limited.

To our knowledge, this study is the first to identify urban residence as an independent predictor of 1-year survival in elderly AML patients receiving decitabine. This likely reflects disparities in access to early diagnosis, hematology care, and supportive services. In Vietnam, rural patients often face logistical barriers, low health literacy, and delayed care-seeking, leading to more advanced disease at presentation. Prior studies have linked rural residence with unmet healthcare needs and reduced service utilization among the elderly [9]. Urban residence may serve as a proxy for better access and care continuity. These findings underscore the need for integrative oncology–public health research, especially in LMICs where geographic and systemic disparities may influence leukemia outcomes.

In the multivariate model, higher baseline BMCc was independently associated with increased 1-year mortality. BMCc represents the total nucleated cell count in the bone marrow aspirate, including leukemic blasts, intermediate progenitors, and mature hematopoietic cells. Unlike the bone marrow blast percentage, which reflects leukemic burden alone, BMCc captures total marrow activity, including both malignant and residual hematopoietic components, thereby serving as a more integrative marker of disease aggressiveness. While previous studies reported inconsistent associations between blast counts and prognosis [7,10,11], this is the first study to identify BMCc as a prognostic factor in elderly AML patients treated with decitabine. Notably, neither marrow nor peripheral blast percentages predicted survival in our cohort, suggesting that BMCc may offer complementary prognostic value. Given its availability from standard aspirates without additional testing, BMCc represents a practical biomarker for risk stratification in resource-limited settings. Further validation is warranted in larger, multicenter studies to confirm its utility in guiding treatment decisions.

In this study, elevated baseline fibrinogen was independently associated with increased 1-year mortality (HR: 1.26, 95 % CI: 1.03–1.54). As a liver-synthesized coagulation factor and acute-phase reactant, fibrinogen rises in response to systemic inflammation, infection, or tissue injury, conditions frequently seen in AML. A meta-analysis by Zhang et al. (*n* = 2947) also linked elevated fibrinogen to a 21 % higher mortality risk in AML patients [6]. These findings suggest that fibrinogen may reflect disease burden and inflammatory status, supporting its role as a simple, accessible prognostic marker. Incorporating fibrinogen into AML risk models could improve risk stratification, especially in low-resource settings. Further studies are needed to validate its predictive value and elucidate underlying mechanisms.

Moreover, elevated baseline urea levels were independently associated with increased 1-year mortality (HR: 1.18; 95 % CI: 1.03–1.35; *p* = 0.015). As a routine renal function marker, urea may reflect subclinical kidney impairment or systemic catabolic stress, both relevant in elderly AML. Although patients with overt renal failure were excluded, subtle dysfunctions may still influence outcomes. Notably, while both urea and creatinine were associated with survival in univariate analysis, only urea remained significant in multivariate modeling. Compared to creatinine, which is affected by muscle mass, hydration, and age, urea may better capture early renal stress and physiological burden. Prior studies have linked elevated urea and blood urea nitrogen to poorer leukemia prognosis [12,13]. These findings support the use of urea as a simple, widely available and inexpensive biomarker for early risk stratification in elderly AML, particularly in low-resource settings.

Among immunophenotypic markers, CD64, a monocytic-lineage marker that delineates AML with monocytic components (M4/M5), showed an independent association with improved 1-year survival in our decitabine-treated cohort. It has been reported as a favorable prognostic marker in several studies. For instance, Zhu et al. found significantly longer OS in CD64-positive patients [8]. In our cohort, CD64 was not significant in univariate analysis but emerged as protective in the multivariate model, highlighting the importance of covariate adjustment in revealing independent prognostic signals. Mechanistically, decitabine has been shown to induce myeloid and monocytic differentiation through epigenetic hypomethylation and FOXO3A-mediated transcriptional activation [14,15]. Although these findings provide a biological rationale for differentiation-linked responsiveness, clinical evidence connecting monocytic phenotype to decitabine efficacy remains limited and heterogeneous among AML patients*.* As CD64 is routinely assessed in standard flow cytometry panels, it may represent a feasible and clinically relevant biomarker for risk stratification in resource-limited AML settings. Prospective validation in larger cohorts is warranted.

In this study, elderly AML patients received a median of four decitabine cycles, with a median OS of 276.0 days - comparable to prior reports in similar populations [10,16]. The 1-year OS rate was 38.6 %, consistent with a multicenter prospective study from China [2]. Among 70 enrolled patients, 39 (55.7 %) completed ≥4 cycles; of these, 48.7 % achieved complete remission (CR). However, no significant OS difference was observed between those who achieved CR and those who did not, aligning with findings from Park et al. [16]. Since response could only be evaluated in patients who completed ≥4 cycles, including it in survival models would introduce selection bias. By analyzing predictors available at diagnosis, the study provides practical insights for early risk stratification in real-world settings, where early mortality and treatment discontinuation are common. The lack of OS difference between responders and non-responders likely reflects the limited durability of decitabine responses and the impact of early mortality or treatment discontinuation in frail elderly AML. While CR remains the ideal endpoint, HMAs may confer survival benefit through disease stabilization and hematologic improvement rather than CR per se [16].

Despite the relatively small sample size, the final Cox model incorporated only five clinically relevant variables and demonstrated acceptable performance, supported by internal validation (*C*-index and cross-validated Brier score). All predictors remained statistically significant. Methodological studies support the use of well-specified models with events per variables below 10 when internal validation is applied [17]. Importantly, this study provides rare real-world evidence from a resource-limited LMIC setting, where routine access to molecular diagnostics and comorbidity assessments is limited. The identification of simple, accessible prognostic markers may facilitate early risk stratification and guide supportive care in similar clinical contexts. Larger multicenter studies are needed to confirm these findings and assess additional molecular predictors.

## 5 Conclusions

This first prospective study of elderly Vietnamese AML patients treated with decitabine identified baseline clinical, hematologic, and immunophenotypic predictors of 1-year survival. Elevated BMCc, fibrinogen, and urea levels were associated with increased mortality, while CD64 positivity and urban residence predicted better outcomes. These findings highlight the prognostic value of accessible markers for early risk stratification in resource-limited settings. Further multicenter studies are warranted to validate these indicators and support their integration into standardized prognostic models for elderly AML patients receiving low-intensity therapy.

## Financial disclosure statement

This research did not receive any specific grant from funding agencies in the public, commercial, or not-for-profit sectors.

## CRediT authorship contribution statement

**Ha Thanh Nguyen:** Writing – original draft, Methodology, Investigation, Formal analysis, Data curation, Conceptualization. **Quoc Khanh Bach:** Writing – review & editing, Validation, Methodology, Formal analysis, Conceptualization. **Quoc Nhat Nguyen:** Writing – original draft, Methodology, Investigation, Formal analysis, Data curation, Conceptualization. **Van Nam Nguyen:** Writing – review & editing, Formal analysis, Data curation. **Thi Van Anh Nguyen:** Writing – review & editing, Validation, Methodology, Formal analysis. **Hai Pham-The:** Writing – review & editing, Formal analysis, Data curation.

## Declaration of competing interest

The authors declare that they have no known competing financial interests or personal relationships that could have appeared to influence the work reported in this paper.
